# Exploring the cultural perspectives and implications of infertility among couples in the Talensi and Nabdam Districts of the upper east region of Ghana

**DOI:** 10.1186/s40834-023-00225-z

**Published:** 2023-04-19

**Authors:** Anthony Kolsabilik Kuug, Sindiwe James, Jardien-Baboo Sihaam

**Affiliations:** 1 Department of Nursing, School of Nursing and Midwifery, University of Health and Allied Sciences, Ho, Ghana; 2 Department of Nursing Science, Nelson Mandela University, Port Elizabeth, P O Box X77000, Gqeberha, 6013 South Africa

**Keywords:** Infertility, Culture, Couples, Ghana

## Abstract

**Background:**

Infertility remains a major clinical and social problem, affecting approximately one in every 10 couples. It is a reproductive health condition that is silently experienced with deep repercussions in the essence of self. In Ghana childbearing is considered a social prestige, where the couples are unduly pressurized to bear children for purposes of genealogical continuity.

**Purpose:**

This study explored the cultural perspectives and implications among males and females experiencing infertility in the Talensi and Nabdam districts of the Upper East Region of Ghana.

**Methods:**

This study used an ethnographic design to explore the perspectives of couples on socio-cultural beliefs about infertility on 15 participants made up of 8 male and 7 female couple units. Participants were selected using a purposive sampling technique while semi-structured interviews were used to explore the cultural implications on male and female couple units. The data were analysed using Tesch’s method of analysing qualitative data.

**Results:**

Two broad themes and five subthemes emerged from the data analysis regarding the cultural implications of infertility. The major themes and subthemes include: (1) Varying cultural perceptions of infertility (Cultural beliefs and perceptions regarding causes of infertility, cultural consequences of infertility and traditional remedies for infertility), and (2) family dynamics stemming from infertility (abuse from family members, and parenthood as a standard for family inheritance).

**Conclusions:**

This study adduces evidence of the cultural implications of infertility in rural Ghana. Considering the cultural inclination of most Ghanaian communities, especially the current study setting, it is imperative that policymakers and public health practitioners should consider fertility interventions that are culturally sensitive. Also, culturally sensitive intervention programs that are targeted at increasing the awareness of the rural population on fertility and its treatment should be considered.

## Background

Infertility remains a major clinical and social problem, affecting approximately one in every ten couples worldwide [1]. Beyond physiological impediments or ‘physical’ causes of infertility, there are also some psychosocial and cultural consequences to ponder [2]. Some researchers have argued that, although infertility is normally identified at first as a physical problem, it comes with a broad and holistic experience commonly associated with psychological, emotional, and spiritual distress [3].

Globally, infertility poses devastating consequences to married individuals regardless of which partner of the union is affected especially in societies where the ultimate reason for marriage is to have children [4]. In a typical African society, fathering a child is a fulfilment of a role that every male hopes to achieve, especially in marriage [5]. In that regard, the African male in marriage is always under intense pressure to prove his manhood. In the same vein, motherhood is celebrated in some communities in Africa, especially in the Upper East Region of Ghana, if a woman is able to bring forth a certain number of children in most cases eight children or more [6].This is similar to ‘Badudwan’, concept among the ‘Akan’ tribe in Ghana to award couple who deliver up to 10 children because of epidemics that killed most people in the past [7].Thus, the importance attached to parenthood makes infertility undesirable in marriages and could sometimes be a major cause of divorce. However, in some instances, both the male and the female are stigmatised, but the culture allows the men to de-stigmatise themselves from the condition by opting out of the marriage or re-marrying [8–10]. In Upper East Region, most people believe and practice patrilineal inheritance. As a result, the inability of partners in a union to reproduce offspring for the purposes of inheritance, results in pressure on couples to conceive, leading to marital instability, depression, low self-esteem, amongst many factors [6]. Some studies in Ghana report that, in most communities, infertile individuals are sometimes denied proper burial at death and denied family property, including land for farming purposes [6, 11].

Even though several studies about infertility have been conducted in the northern and southern sectors of Ghana, it is important to note that every region or district has sub-cultures, which makes the experiences of infertility differ significantly in the different communities and settings [5, 6, 12–15]. Even though some qualitative studies have been done in the Upper West Region and North East Regions [6, 16], their focus was on the experiences and effects of infertility among women. None of the studies on infertility in the Northern regions has considered the experiences of men with infertility. Also, a study by Ofosu-Budu and Hanninen [16] considered the cultural perspective of infertility in one of the districts in North East Region but this cannot be generalised because of cultural diversity of the Northern Regions of Ghana. In West Mamprusi District in the North East Region which is a polygamous society, a man easily re-marries when he is faced with infertility while contravening marital vows by seeking for pregnancy outside marriage is common with people from the Talensi and Nabdam communities. Also, humiliating the dead by burying them in isolated lands as well as piercing their reproductive organs with thorns is a common practice among the Talensi’s while such practices are not common in the Muslim dominated societies in North East Region [6, 8, 16].

This study, therefore, sought to explore the cultural perspectives and implications of infertility on males and females separately and the implications for infertility management in the Talensi and Nabdam districts of the Upper East Region of Ghana. Currently, there are no guidelines addressing infertility management, however, infertility services are incorporated into Ghana Health Service (GHS) reproductive health policy. Therefore, this study will direct and shape health education toward effective management and reducing the psychological trauma of infertility in Ghana and beyond.

## Materials and methods

### Study design

This study used an ethnographic design [17] to explore the perspectives of couples on socio-cultural beliefs about infertility. A focused ethnography was much suited for the study as the researchers sought to explore socially and culturally diverse beliefs with intense data collection and less time spent in the field [17, 18]. This design also allows the researcher to discover and understand a phenomenon, a process or the perspectives and worldviews of the people involved [19]. This design is very relevant, especially where information is required directly from those experiencing the phenomenon under consideration, in this case, cultural implications of infertility amongst males and females [20]. This study considered the subjective views on the cultural implications of infertility among male and female participants.

### Study setting

The study was conducted in the Talensi and Nabdam districts of the Upper East Region. The Upper East Region of Ghana comprises thirteen districts, of which Talensi and Nabdam are inclusive. People from the Talensi and Nabdam districts are predominantly farmers, who adhere to traditional and cultural beliefs and values making health education programmes a challenge. There is a high illiteracy rate among the people in the region [21]. The Upper East Region has only one regional hospital with the rest of the health facilities being district hospitals, health centres or clinics, and therefore lacks fertility hospitals or clinics to offer infertility services. The fertility rate of the region per Ghana Statistical Service (2015) stood at 4.5% while male infertility was discovered as an emerging reproductive health concern in the region [21].

### Sample and sampling technique

Purposive criterion sampling was used to select male and female partners in marriage who experienced infertility and were willing to participate in the study which included couple units with primary infertility who had been married for more than two years without successful impregnation of their female partners. The couples must have stayed together continuously for two years or more and should not have been practicing any contraception. The study excluded couples with secondary infertility. Participants who presented with infertility concerns were selected purposefully at the Out Patients’ Department (OPD) of the various health facilities in the districts through the information obtained during history taking and followed up to their homes for recruitment. Men who accompanied their wives for infertility evaluation and women who had their husbands’ consent and were willing to participate were included in the study. Also, with the help of village health volunteers’ participants were traced to their homes for the necessary consent processes.

### Data collection

Fifteen (15) Participants (8 males and 7 females) were purposefully recruited from the health facilities and communities in the study area, after having consented to be part of the study. Data was collected between September 15th 2019 and December 16th 2019. Males and females as willing individual partners in a couples’ unit – took part at separate venues in sharing their experiences of their culture on infertility. Participants experiencing infertility were not interviewed as a couple-unit with their partners, but separately as individuals. This enabled the researcher to get the unique male and female perspectives of their culture on their infertility experiences. Data were gathered through semi-structured interviews by the lead author (AK) using an interview guide to direct the interactions. The interviews were conducted on participants’ own time either in the comfort of the participant’s residence or at an agreed venue in order not to disrupt their daily activities. These questions were asked in the native language and the responses of participants were digitally captured using a voice recorder for transcription. Participants were allowed to freely share their experiences. Each interview lasted for at least 45–60 min. The number of participants was determined by data saturation. Data saturation was achieved at the 15th participant when no new information was obtainable from the participants.

### Data analysis

Recorded interviews were transcribed into English for analysis. Using Tesch’s method of analysing qualitative data [22], the eight-step data analysis process cited in Creswell [20] was applied by the researcher which includes reading the data thoroughly, clustering similiar topics and abbreviating them as quotes, finalising the decision on each quote and recoding where necessary. An independent coder was used to enhance the trustworthiness as well as the credibility of the results obtained. Two broad themes and four sub-themes emerged.

### Ethical consideration

Approval for this study was sought from the Navrongo Health Research Centre Institutional Review Board (NHRCIRB) with protocol version H18-HEA-NUR-003. Participants’ consent was sought verbally by explaining the importance of the study to them and their role in the research process to gain their cooperation and understanding. Participants who agreed to participate in the study were informed of their right to withdraw from the study at any point in time if they wished to.

### Trustworthiness

The methodological rigor was ensured following the criteria outlined by Lincoln and Guba which include credibility, dependability, conformability, and transferability. They, later on, included authenticity [23]. To ensure credibility the researchers observed the physical environment, identified areas of interest during the interview, interviewed males and females separately, and used member and peer checking and debriefing. Dependability was ensured through peer debriefing and maintenance of the process log to ensure uniformity of the research process [24]. Conformability was ensured by keeping an audit trail of the methodological process. Transferability was ensured through a thick description of the cultural beliefs and perceptions regarding infertility. Authenticity was ensured through the selection of the appropriate participants for the study with a rich and detailed description [23, 24].

## Results

Eight (8) males and 7 females from the Talensi and Nabdam communities were interviewed. They were mostly farmers or traders and their ages ranged from 29 to 52 years as shown in Table 1.

**Table 1:** Demographic profile of Participants

Number	Participant label (PL)	Age (yrs)	Gender	Occupation	Years in marriage without a child	District/Community
1.	001	33	M	Farming	5	Nabdam (Gari)
2.	002	52	F	Farming	26	Nabdam (Pelungu)
3.	003	33	M	Teaching	6	Talensi (Tenzuk)
4.	004	40	M	Farming	7	Talensi (Tenzuk)
5.	005	45	F	Farming	8	Naddam (Datuku)
6	006	35	M	Trader	6	Nabdam (Garizoo)
7	007	38	M	Farming	7	Nabdam (Tindongo)
8	008	32	F	Trading	6	Talensi (Baare)
9	009	40	F	Trading	9	Talensi (Winkongo)
10	010	41	M	Farming	8	Nandam (Kongo)
11	011	29	F	Farming	6	Talensi (Tongo)
12	012	35	M	Farming	6	Talensi (Yindore)
13	013	29	F	Trader	5	Nabdam (Gari)
14	014	42	M	Farmer	7	Nabdam (Tindong)
15	015	39	F	Trader	9	Tanlensi (Yahzoore)

From the data analysis two major themes emerged with five subthemes. The major themes and subthemes include: (1) Varying cultural perceptions of infertility (Cultural beliefs and perceptions regarding causes of infertility, cultural consequences of infertility and traditional remedies for Infertility) and (2) family dynamics stemming from infertility (abuse from family members and parenthood as a standard to qualify for family inheritance).

### Varying cultural perceptions of infertility

This study found varied cultural beliefs and perceptions regarding infertility among females and males. Participants voiced their frustration for being perceived as witches and for that reason are punished by the ‘gods’. Some said they are considered ‘cursed’ individuals and others were accused of promiscuity.

#### Cultural beliefs and perceptions regarding causes of infertility

The perceptions, as reported by the participants, are mainly related to cultural differences. Some think infertility is linked to witchcraft. Some believe that infertility is a punishment for being promiscuous in life. Participants also enunciated that infertility was a curse and a punishment from the gods and ancestors.

A participant had this to say:

*”I was accused in the market for attempting to transfer my witchcraft to a young boy I sent. The mother rained insults on me and said my witchcraft has left me childless so I should leave her son alone” (IDI-002 Female).*

Others say it is caused by being promiscuous and this was a quote from a participant:

*“The community members see those of us without children as being punished for being promiscuous even including our wives… Some say we cannot have children because of our promiscuous lifestyle” (IDI-007 Male).*

While some think it is a spiritual curse, others think it is a punishment from the gods and disapproval of the marriage by their ancestors. Regarding the perception of infertility, one female participant had the following to say:

*“In this community, infertility is associated with so many factors but the most common cause of it in this community is disapproval of the marriage by our ancestors...” (IDI- 008 Female).*

Another reported infertility as a generational curse as presented below:

*“As for me, I am a traditional man, so I have been to a soothsayer who said we need to perform some sacrifices to break some generational ‘curses’ so, in addition to the herbs, I am doing those sacrifices too...” (IDI-006 male).*

#### Cultural consequences of infertility

Individuals believed to be cursed are stigmatised and are normally isolated. Participants were viewed as being cursed due to infertility and were at times told so or removed from the community. Participants indicated that community members dissociate themselves from infertile people. In expressing these experiences of being perceived as cursed, participants had the following to say:

*“They see us to be evil and cursed by the gods so people are afraid to associate with childless men because they can also be punished for associating with the ‘cursed’” (IDI-001 Male).*

…they say childless men are cursed… (IDI-003 Male)

*“We are perceived to be cursed by the gods for some wrongdoings and probably not useful to society that is why the gods do not want us to procreate … Some say we are witches and cursed by the ancestors or gods for not being good members of the society...” (IDI 009 Female).*

*“Infertility is seen as a curse and a form of punishment, so no one wants to associate with us. Apart from seeing us as being strange, some avoid us completely...” (IDI-013 Female).*

The male partners expressed anxiety due to being pressurised by family members to make their wives conceive children, which was further compounded by the increasing age of these women, as childbearing follows a ‘biological clock’ (that is there is a time limit of being biologically capable of giving birth).

There is an increased risk associated with pregnancies and outcomes in women with higher maternal ages, so husbands (males) turn to be worried. This is one of the quotes from a worried participant:

*“As I speak to you right now, I don’t even know whether my wife can still make babies for me. We have tried all forms of treatment to no avail and the more we try to make babies, the more the pressure keeps mounting from close family members and friends” (IDI 001 Male).*

Socially and culturally, male couples in the Talensi and Nabdam districts who have no children are faced with social ridicule, scorn, and isolation, as attested to by most participants. They are treated inhumanly at death, such as being buried on isolated lands for fear that they may make the lands infertile and piercing their genitals after death as an indication that the organs were not used to produce children whilst they were alive. Participants were worried about life after death and what would happen to their bodies when they die. Participants who were worried about how they would be buried said:

*“There is this place very far away from the family lands meant for the burial of outcasts, and barren couples. It is believed that when buried on or near family land, the land will become infertile, and anything done on such lands will not flourish. This practice brings a lot of shame and disgrace to our families. In death also, men and women are dressed differently for burial and the number of days for performing the final funeral rites also differ. For instance, as a man without a child, when I die, I will be dressed as a woman and the duration of my funeral will be that of the woman because without a child you are considered a woman” (IDI-012 Male).*

*“Apart from the fact that I am to be buried far away on an isolated land where no one goes, my genitals would be mutilated or pierced with a thorn before burial. It is believed that if they do not do that, I can go after other people’s wives in the spirit world to get the children that I could not have on earth. This is so humiliating that relatives of the deceased are not able to overcome it” (IDI-010 Male).*

Sometimes, infertile people are denied family inheritance, such as land and other property, and women who lose their spouses are denied the same and sometimes even driven away from their matrimonial homes. A participant who expected to inherit his father’s property was told by his kinsmen that he could not because of his childlessness. He had this to say:

*“I was once told by my family members that the farmlands my father left for me were going to be taken and given to other family members who have use for it because the lands were too much for a man without a child to feed or inherit him,” They said that as a man, I cannot inherit my father’s property including the gods even now that he is dead and what kind of a man am I?” (IDI-003 Male).*

#### Traditional remedies for infertility

Participants described the various traditional remedies for infertility based on their cultural beliefs. This was seen based on the participant’s expressions. A male participant expressed his frustration on what is termed ‘the weird cause of infertility’ or ‘punishment from the gods’. He described the solutions for infertility by the community members as traditional, since it is believed that the cause is a form of the curse by the ancestors. He said:

*“My wife had to go through the traditional sacrifices to appease the ancestors… That is a cultural belief here and which is held so high than any form of treatment” (IDI- 010 Male).*

Some of the participants, regardless of their level of education, still perceived infertility to be caused by some supernatural forces but not medically related. Such an experience is noticed in the response of one of the participants, who is a teacher:

*“In this community, infertility is the result of curses from our ancestors, which requires spiritual cleansing” (IDI 003 Male).*

One of the participants who believed that the condition had some supernatural underpinnings, is quoted as:

*“We have tried several times using different modes of treatment but have not succeeded. Our family head compelled us to make traditional sacrifices with a ram, a white cock, cola nuts, cowries and some drinks to the gods as they believe infertility is spiritually inflicted on people” (IDI-005 Female).*

Experiences expressed by men with infertility largely reflected the cultural and traditional values. Their beliefs largely influenced the preference for other forms of treatment rather than scientific medicine. One of the participants said:

*“I am looking for help from other sources of treatment. We are doing herbal treatment and currently, my sister got us some herbal preparation which she says has helped so many people … They say it is not good to put all your eggs in one basket, so I am doing the ‘black’ medicine in addition. I also keep offering sacrifices regularly per our customs here, so that if we have offended the gods in any way, they will have mercy on us” (IDI-014 Male).*

Similarly, some participants believed that infertility management is effective using traditional and unorthodox forms of treatment. One participant shared his belief regarding how infertility had spiritual and traditional undertones and his efforts at getting a solution:

*“In order to overcome any perceived ‘curse’ as believed by the people here, I have spent more time seeking treatment in various forms. I have been to my pastors for prayers, I have been to herbalists who gave some preparation for me to take for three months and my wife has equally performed sacrifices but pregnancy never came” (IDI-004 Male).*

In this study, it was evident that respondents were tied to their cultural beliefs that infertility treatment should also include herbal preparations of various forms. This is what one participant had to say:

*“I have taken the local medicine before for any ‘curse’ and I have taken herbal preparations also from herbalists but still expecting results” (IDI-007 Female).*

### Family dynamics stemming from infertility

Constant pressure from family members for couples to conceive immediately after marriage has led to some of them maltreating, abusing, and humiliating individuals who have challenges conceiving.

#### Abuse from family members

Such concerns also emerged in this study, for example, some male participants said they were constantly being abused and humiliated by either their mothers-in-law or other members of the family and community. Responses such as those below were given:

*“As soon as my mother-in-law sets her eyes on me, no matter what time of day or night it is, she would start to abuse me verbally … She keeps asking me to leave her daughter alone so that she can get her a ‘real’ man who will give her twins within one year” (IDI-014 Male).*

“My mother-in-law *can rain insults on me the whole day, calling me an incompetent man. Can you blame her? Every woman will certainly want to be called a grandmother” (IDI-014 Male).*

According to participants, they experienced humiliation and loneliness when they returned from farms to their empty homes and wished they could be attending to their children as other couples do. This is what one of the participants had to say:

*“I sometimes feel like staying on my farm the whole day instead of coming home because of the humiliations I get from family members. You can imagine when everyone is happy with their children after the day’s work, and I stay here alone with my wife lonely” (IDI-014 Male).*

#### Parenthood as a standard to qualify for family inheritance

In African traditional societies, the reasons to have children comes with a strong cultural background and the perception that males in marriage will always strive to meet this cultural or traditional expectation. Consequently, in this study, similar perceptions were noted, as one participant, for example, explained his perception of marriage in a patrilineal society:

*“Every man in marriage in our community aspires to fulfil a fatherhood status of having children, especially male children, to succeed him at old age and death … You see, in this part of the country, childbirth is so important to our men because of the patrilineal inheritance, and for that reason, a man without a child has no one to inherit him when he dies and so the family lineage is terminated” (IDI-007 Male).*

Male participants who had been married for eight and ten years respectively without a child and who were stressed by the circumstances had similar perceptions in this regard and the following to say:

*“Because of the patrilineal inheritance system, every man wishes to have children to succeed him after retirement or death, especially male children. So, if you even give birth to female children, they still consider you not to have given birth appropriately. When it comes to inheritance, the first male child is supposed to be the heir to his father’s property. ‘This, however, is only possible if you have children. If not, you are stripped off all these rights and given to your younger siblings” (IDI- 006 Male).*

Infertility thus becomes a concern as it causes neither of the aforementioned expectations to be fulfilled and therefore puts a strain not only on the males in marriage but also on the couple as a whole.

## Discussion

This study explored the cultural perspectives and implications of infertility on males and females and the implications for infertility management in the Talensi and Nabdam districts of the Upper East Region of Ghana. The study provides evidence on the reasons why individuals want parenthood as well as the cultural implications that accompany childlessness in rural communities, such as the family and societal pressure. Though cultural, societal, and family pressure on infertile couples’ cuts across the country, the magnitude differs due to cultural diversity [6, 12, 16, 25]. For instance, while the upper East Region practices a patrilineal system of inheritance thus placing importance of male children [26, 27], people from some part of southern Ghana practice matrilineal system of inheritance so couples with infertility may not place emphasis on male children even though the pressure to bear children is same for all regions in Ghana. These cultural differences are seen in the forms of religion people practice. For instance, among residents of West Mamprusi District where polygamy is a common practice, males with infertility find solace in taking a second wife [8] while couple with infertility in the Talensi and Nabdam Districts will permit the woman to ‘experiment’ with another man in the family to achieve pregnancy. Also, the belief in supernatural causes of infertility could stem from the fact that a greater proportion of the people of the Upper East are fixated with their cultural beliefs and norms where the consequences and stigma of infertility even linger on in death.

Also, stigmatisation came across as a major demotivating factor for individuals with infertility.

According to a study by Martins et al., [28], the belief by some members of traditional societies that infertility occurs as a result of contravention in terms of the ancestors, consolidates the assertion that anyone with infertility is cursed. Therefore, once the cause of infertility is diagnosed as traditional, the remedy has to be traditional to appease the gods and the ancestors before the curse can be reversed [15]. When people’s culture is an integral part of them, it becomes difficult to get them to understand issues of reproduction from a different perspective. Participants in this study reported the effect of culture on infertility as a traditional ‘curse’ to have influenced their lives. The health-seeking behaviour of any society or a group of people depends among other factors, on what is thought or perceived to be the cause of that condition according to the health belief model [29]. This also goes to explain that, depending on the cause of a condition, appropriate solutions are sought to bring relief to those afflicted according to their beliefs.

Similarly as reported in sub-Saharan Africa, women primarily bear the brunt of infertility because their male partners dissociate themselves from the condition [10]. The women are therefore mostly left to find remedies for the infertility even if they might not be the cause of the infertility. In some communities in Zimbabwe, the subject of male infertility is discussed in secrecy, and most men do not want to talk about it, let alone avail themselves for evaluation and treatment [30]. Though in the current study, the male partners also bore the brunt of infertility, the societal and family pressure were much on the females than the males. The males have the opportunity to go in for another wife in order to have children while the women are left in an uncertain state or will likely face divorce due to their inability to bring forth children. Most women therefore face cultural and social ridicule due to the infertile state they find themselves. In most recent times the number of couples experiencing infertility is reportedly increasing and the social stigma of childlessness persists and is becoming more challenging for these couples, as it interferes with treatment outcomes [31]. A related study, found that stigma and discrimination reflect an erroneous perception of the condition and these had led to the poor management and treatment of the couples experiencing infertility, especially in sub-Saharan Africa [32].

Infertility comes with much-associated stigma and concern for its management in terms of physical, psychological, emotional and financial problems [31]. With regards to the cultural implications, couples living with infertility mostly suffer humiliation and public ridicule and are excluded from cultural or social functions [14]. Couples in this study were stigmatized as their condition was attributed to past misdeeds, they being evil, and past promiscuous lifestyle. Consistent with the current study findings, couples in Zambia who were infertile were faced with public ridicule not only during their lifetime but also after death [33]. Couples in this current study face inhumane treatment after death due to infertility such as being buried in isolation and their genitals pierced. This treatment meted out to infertile couple after death had a huge psychological effect on couple. Participants revealed that, apart from not being welcomed at important family gatherings, they were also not allowed to mingle with strangers or handle new-born babies, especially the female’s experiencing infertility.

Congruent to this study finding, results in a study conducted in a rural society in Zimbabwe revealed that interventions for infertility were exclusively traditional which comprised two forms [30]. The interventions comprised either administering aphrodisiacs in the form of tree barks and roots mixed with porridge, or consulting with traditional healers for divination purposes and subsequent traditional treatment [30]. It is interesting to note that the majority of the participants revealed that their first point of call for solutions for infertility was not the hospital, but unorthodox medicines and other forms of treatment [34]. Most of them believed in religious activities as the first consideration in finding solutions for infertility, especially to break any generational curse.

Parenthood in Africa is regarded very highly because of the value that children bring to their parents in society [5]. For instance, children are seen as symbols of pride and prestige for parents, a source of hope for the future, a guarantee for family lineage as well as inheritance, and sometimes as a source of labour for their parents [8, 35]. In some instances, children in Africa are also considered a form of fulfilment and insurance for parents in their old age [36]. In the Upper East Region of Ghana, children are also used to measure a man’s wealth among his kinsmen, and thus, infertility becomes stigmatised. It is more revealing to understand that the stigma accompanying infertility is not only present when couples are alive, but also persists even after their death, so no one is expected to be associated with infertility [8, 25, 37].

In a traditional community like the Upper East Region of Ghana, leadership roles are reserved for men with children [6, 11]. In a study conducted in Nigeria on the cultural constructions of infertility, it was found that men without children cannot be clan heads, neither can they be opinion leaders nor be considered for chieftaincy [38]. In a related study in Zimbabwe, a couple without children are not allowed to take up leadership roles in their respective men’s or women’s groups [30]. For these reasons, couples in the Talensi and Nabdam districts will do anything – including accepting to contravene their marital vows such as having secret relationships with other people in orderr to have children – to secure their marriages, especially giving birth to male children.

In the light of cultural and traditional beliefs, childbearing in this study was viewed by participants as an important asset and social recognition. In the Ghanaian cultural perspective, property acquired by couples, or the family members are passed on as inheritance to the children, so giving birth is considered very vital, therefore families strive to acquire wealth for their children. Following one’s inability to have children in some Ghanaian cultures, the individual is denied of family inheritance especially women which was widely enunciated by many participants. Even though the current Ghanaian laws regarding inheriting the property of a deceased spouse is clearly spelt out, victims who are mostly women without formal education are not informed regarding what their rights are. This should be considered to inform health education strategies. The social prestige and recognition of childbearing as gift and the value that participants placed on children in this study is consistent with previous studies in Ghana [11, 25, 39]. Considering the cultural inclination of most Ghanaian communities especially the current study setting, it is imperative that policymakers, and public health practitioners should consider fertility interventions that are culturally sensitive.

Some families faced with infertility tend to get closer to offer support, compassion and understanding amidst the highly anxious situation [40]. However, in some typical rural African societies, such as the Talensi and the Nabdam communities, there is little or no support for couples experiencing infertility. Our finding is inconsistent with some studies where couples expressed psychological/financial support from partners/family members in the time of childlessness [3, 40]. Evidence indicates that even if family and other loved ones fail to provide adequate and positive support, the partner’s empathy, and affection, with adequate support provides the necessary emotional support for the partner to keep hope alive and continue the fertility treatment [3]. We propose that social support systems for couples with infertility in rural Ghana should be established in order to boost the emotional and mental state of couples facing difficulty in having children. Also, support systems that consider introducing infertile couples to each other and those that have passed through this condition and have given birth successfully is imperative.

A recent study in Nigeria revealed that the first choice of treatment for infertility problems by couples was the hospital [40]. Participants in the present study sought for traditional remedies for the management of infertility instead of the orthodox form of treatment. The possible explanation for these findings is that the majority of the present study participants were peasant farmers and traders while the majority of participants in the Nigerian study had tertiary education, and this might have influenced their health seeking behavior. We recommend that public health education and sensitization on infertility and its treatment should target rural communities including those without formal education. Also, culturally sensitive intervention programs that are targeted at increasing the awareness of the rural population on infertility and its treatment should be considered.

### Implications for nursing and midwifery practice

This study adduces evidence on the cultural implication of infertility on male and female couples in the rural settings of the Upper East region of Ghana. The cultural beliefs of infertility within the traditional communities had a negative effect on couples as they were stigmatised. The institutionalisation of fertility/infertility programs in rural communities to create much awareness and possible remedies for infertility would be imperative to reduce the social stigma associated with childlessness. Social and family support for couples with infertility would be very vital as this will go a long way to reduce the psychological and emotional distress associated with the condition as couples with infertility are faced with social and family pressure to produce children. Education of partners affected by infertility regarding the laws governing inheriting the property of one’s spouse (the interstate succession law) should be enforced. As infertility, a serious reproductive health challenge was much associated with curses and contravention of the ancestors, which led to most couples seeking for cultural and traditional remedies, we recommend public health education programs that seek to educate the public on various management methods of infertility be made available.

### Limitation of the study

The findings of this study cannot be generalised to other districts in Ghana. The experiences of males and females regarding infertility in other regions where different cultures exist could have also been explored to understand holistically how culture affects couples experiencing infertility, but due to the scope of the study and resource constraints, that was not possible. The current study can therefore only be understood in the context of the Talensi and Nabdam people. In addition, constraints regarding the scope of the study, time and resources did not permit the researcher to explore the experiences of community members, especially opinion leaders and family heads, to ascertain their views on infertility as that would have enriched the findings of the study.

## Conclusions

This study adduces evidence of the cultural implications of infertility in rural Ghana. Considering the cultural inclination of most Ghanaian communities, especially the current study setting, it is imperative that policymakers and public health practitioners should consider fertility interventions that are culturally sensitive such as health promotion activities that are not based on assumption, but on effective engagement, support for clients and respect for cultural beliefs. Also, culturally sensitive intervention programs that are targeted at increasing the awareness of the rural population on fertility and its treatment should be considered.

## Data Availability

Data is available upon a reasonable request to the lead author.
